# The Relationships of Team Role- and Character Strengths-Balance With Individual and Team-Level Satisfaction and Performance

**DOI:** 10.3389/fpsyg.2020.566222

**Published:** 2020-11-30

**Authors:** Fabian Gander, Ines Gaitzsch, Willibald Ruch

**Affiliations:** ^1^Department of Psychology, University of Zürich, Zurich, Switzerland; ^2^Clienia Littenheid AG, Littenheid, Switzerland

**Keywords:** character strengths, team roles, team role balance, work performance, work satisfaction, teamwork quality

## Abstract

Teamwork has been argued to play an increasingly important role in numerous jobs, and several studies focused on the effects of team composition for work-related outcomes. Recent research has also identified individuals’ character strengths and positive team roles (e.g., idea creator and relationship manager) as conducive to work-related outcomes. However, there is a scarcity of research on the role of character strengths or positive team roles on the level of teams. In the present study, we extend theoretical assumptions of team role theories to the study of character strengths and positive team roles: We examined the associations between character strengths and team roles with work-related outcomes on the individual (i.e., job satisfaction, self- and supervisor-rated performance) and the team level (i.e., teamwork quality, self- and supervisor-rated team performance). Further, we examined how the team composition relates to the outcomes, that is, whether balanced teams (i.e., all team roles or character strengths are represented in the current team) go along with desired outcomes and whether an overrepresentation of team roles or character strengths in a team (i.e., a team role or character strengths is represented by multiple team members) goes along with undesired outcomes. We studied a sample of 42 teams (*N* = 284 individuals) who completed measures of team roles, character strengths, teamwork quality, job satisfaction, and self-rated individual and team performance. Further, supervisor ratings of individual and team performance were collected. Results corroborated the relationships of team roles and character strengths with individual outcomes such as that specific roles and character strengths go along with individual performance and work satisfaction. Further, the results suggested that teams in which more team roles are represented report higher performance and teamwork quality. Also, teams with higher average levels of the character strengths of teamwork and fairness, and teams with more members scoring high in fairness and prudence report higher teamwork quality. Further, there is no evidence that having too many members with a particular character strength has detrimental effects on teamwork quality, work satisfaction, or performance. We conclude that extending the study of character to the level of teams offers an important advancement.

## Introduction

Teamwork has often been highlighted as an important factor for the success of projects and organizational performance (e.g., Petty et al., 1995; Hoegl and Gmuenden, 2001). A considerable body of literature has focused on the composition of successful teams, and several relevant factors for successful teamwork have been proposed. A meta-analysis reported the diversity of education or expertise within teams to go along with qualitatively better team performance, while no effects for the diversity of demographic characteristics were found (Horwitz and Horwitz, 2007). For other variables such as the personality dimensions of the five-factor model, findings were mostly mixed (see Mathieu et al., 2008 for a review).

However, it has been argued for a long time (e.g., Benne and Sheats, 1948) that diversity (also referred to as balance) in personality-related individual differences, such as team roles, plays a crucial role for performance and work-related well-being of individuals and teams. Recently, a new framework for studying team roles has been proposed, the VIA team roles. This framework has been developed from a positive psychology viewpoint and distinguishes among seven informal team roles that focus on positive behaviors and contributions to the team (VIA Institute on Character, 2013). Initial studies using this framework suggested positive associations between assuming these team roles and relevant work-related outcomes, such as work satisfaction or calling (Gander et al., 2018; Ruch et al., 2018).

Further, within positive psychology, a classification of positively valued personality traits, so-called character strengths, has been suggested (Peterson and Seligman, 2004). This VIA classification encompasses 24 character strengths that are expected to contribute to the “good life” in all its domains. Thus, it is expected that several of these traits also contribute to good work performance and a fulfilling work experience; on the level of individuals, this has been confirmed in earlier studies (e.g., Harzer and Ruch, 2014).

In the present study, we aim at providing some information on how teams could be composed regarding team roles and character strengths in order to maximize desirable outcomes. We extend existing findings by studying complete teams and examine whether the configuration of teams with regard to team roles and character strengths relates to work satisfaction, teamwork quality, and performance.

### Teams and Team Roles

In the present study, teams are considered groups of at least three people who “exist to perform organizationally relevant tasks, share one or more common goals, interact socially, exhibit task interdependencies, maintain and manage boundaries, and are embedded in an organizational context that sets boundaries, constrains the team, and influences exchanges with other units in the broader entity” (Kozlowski and Bell, 2003; p. 334). Team roles are context-dependent behavior patterns (Biddle, 1979) that people display in such teams.

Several conceptualizations of team roles have been proposed (for an overview see Mathieu et al., 2015) with the most influential one suggested by Belbin (1981, 2010, 2012). His framework distinguishes among nine informal roles (i.e., plant, resource investigator, coordinator, shaper, monitor evaluator, team worker, implementer, completer finisher, and specialist). Each of these roles is expected to come along with specific strengths and weaknesses (e.g., coordinators are described as being good at clarifying goals, delegating, and promoting decision making, while also prone to delegating own work to others and being manipulative; Belbin, 2012). Based on this model of nine team roles, Belbin (2010) suggested that teams should be balanced with regard to team roles; that is, all team roles should be present in a team, and no relevant role should be missing, while roles should also not be overrepresented (e.g., duplicated) in a team.

Empirical support for this notion is widely mixed. Several studies reported positive findings; for example, Meslec and Curşeu (2015) found positive relationships between teamwork quality and role balance as a configural group property in a student sample. Senior (1997) also reported supporting evidence for the relevance of team role balance for team performance in a sample of 11 management teams. Other studies failed to find any relationships (e.g., van de Water et al., 2008; Batenburg et al., 2013). Similarly, Meslec and Curşeu (2015) also found no support for the notion that roles should not be duplicated. Overall, results remain inconclusive and research has often relied on very small or student samples. Further, although widely used, Belbin’s model—particularly the associated assessment instrument (Belbin Team Role Self-Perception Inventory; Belbin, 1981)—has often been criticized, mostly for its allegedly unsatisfactory psychometric properties (Furnham et al., 1993a,b; Fisher et al., 2001).

The present study employs a different framework for the assessment of team roles, the VIA team roles (VIA Institute on Character, 2013). It assumes the seven following team roles: Idea Creator (thinks of unconventional ways of coming to solutions and great ideas), Information Gatherer (searches for information, for example, on best practices, new trends, potential vendors, competition, etc.), Decision Maker (processes and integrates available information, makes decisions and clarifies the goals), Implementer (controls the current status and takes measures to work toward the goal), Influencer (presents the product for acceptance internally and/or externally), Energizer (infuses energy into their work and others), and Relationship Manager (helps to run relationships smoothly and to resolve conflicts). These team roles were derived rationally based on considerations about relevant skills following a prototypical sequence in a project: At the beginning, a new idea has to be created (Idea Creator), and research conducted on existing information (Information Gatherer). Then, goals have to be set, and decisions made (Decision Maker), which have to be implemented (Implementer), and internal (e.g., supervisors), and external (e.g., customers) stakeholders have to be convinced (Influencer). Throughout the whole process, obstacles have to be overcome, which requires persistence and energy (Energizer), and a productive work atmosphere has to be maintained, and conflicts among team members have to be resolved (Relationship Manager).

While the VIA team roles share many similarities with Belbin’s approach, they represent a more parsimonious model and exclusively focus on strengths (instead of also entailing weaknesses). Further, a psychometrically sound instrument has been developed for their assessment, the VIA Team-Roles Inventory (Ruch et al., 2018). Nonetheless, several of Belbin’s assumptions are also expected for the VIA team roles, mostly the hypotheses that more balanced teams (i.e., teams in which more of the seven VIA team roles are represented), and teams in which team roles are less overrepresented (i.e., duplicate), should perform better in terms of performance and well-being at work (e.g., Senior, 1997).

Earlier studies showed that all VIA team roles are positively related to individual work satisfaction (Ruch et al., 2018) and calling (with the exception of Information Gatherer; Gander et al., 2018). Further, it has been suggested that the interplay between the team roles one shows in the current job, and the roles one would like to show in an ideal team, also plays a role for job satisfaction: For most team roles (i.e., Information Gatherer, Implementer, Relationship Manager, and partially Idea Creator), a better convergence between current and ideal roles went along with higher job satisfaction. The *levels* of ideal team roles, however, showed only few comparatively small relationships with job satisfaction or calling— in contrast to the levels of team roles actually shown in the current job that were predictive of job satisfaction.

However, currently there is no data available on the relationships between the VIA team roles and work performance. Further, previous studies exclusively relied on self-ratings of individuals and did also not consider teams. Of course, studying configurations of team roles in existing teams and also considering team-level outcomes is of particular importance for advancing the study of team roles and could help in designing well-functioning teams.

### Character Strengths

For studying character, Peterson and Seligman (2004) developed the VIA classification that comprises 24 character strengths (i.e., creativity, curiosity, judgment, love of learning, perspective, bravery, perseverance, honesty, zest, love, kindness, social intelligence, teamwork, fairness, leadership, forgiveness, humility, prudence, self-regulation, appreciation of beauty and excellence, gratitude, hope, humor, and spirituality). For identifying these character strengths, Peterson and Seligman (2004) conducted a comprehensive literature research and applied several criteria (e.g., contributing to fulfillments that constitute the “good life,” being morally valued in its own right, being trait-like, being distinct from other strengths, etc.) to potential candidates for character-relevant traits. In sum, these 24 character strengths represent the predominant model for the empirical study of character.

The relevance of character strengths for work-related outcomes has been emphasized early on. For example, Peterson et al. (2009) suggested that “no matter the occupation, character matters in the workplace (p. 229).” Character strengths have, for example, been shown to go along with well-being at work (Peterson et al., 2009; Gander et al., 2012; Harzer and Ruch, 2015; Heintz and Ruch, 2020; Huber et al., 2020). While usually almost all strengths positively relate to well-being, often, the character strengths of zest, hope, love, gratitude, and curiosity yielded the strongest relationships to both, general and work-related well-being. Further, character strengths are also relevant for work performance: Almost all character strengths predicted self-rated work performance, and several strengths also go along with supervisor-rated performance evaluations, including the strengths of perseverance, teamwork, and honesty (Harzer and Ruch, 2014). Perseverance has been suggested to play the most important role for work performance (Littman-Ovadia and Lavy, 2016).

Further, character strengths have also been linked to team roles. On the conceptual level, Ruch et al. (2018) suggested that “character strengths might guide the preference for certain team roles but also help taking on and performing these roles” (p. 2). On the empirical level, Ruch et al. (2018) showed that some strengths (e.g., zest, teamwork, leadership, and hope) were robustly related to most roles, while other strengths were particularly important predictors for specific roles (e.g., creativity for the role of Idea Creator, social intelligence for the role of Relationship Manager). Thus, team roles and character strengths represent distinguishable, but both conceptually and empirically related concepts. In the present article, we aim at studying the relevance of both concepts in teams separately.

While there is a lot of empirical data on the relationships of character strengths and well-being at work, and a few studies that examined their contribution to work performance, all the studies so far are based on individual data and outcomes. However, since work is rarely conducted in isolation, all real-world settings are also affected by the interindividual interplay of individual differences. Thus, an important next step in the study of character at work is to consider levels and configurations of character strengths in teams, and also to take team-level outcomes into account.

### The Present Study

The present study examined the role of character strengths and team roles for work-related outcomes. Since some previous studies found effects of team role balance on teamwork quality and team performance, and relationships of character strengths with individual performance and work satisfaction, we considered all these variables: We were interested in individual and team-level performance, individual work satisfaction, and teamwork quality (i.e., comprising several aspects of collaborative team processes related to both tasks and social interactions). Further, we considered data from several sources and levels, namely, individual self-ratings, aggregated self-ratings, and supervisor-ratings.

The outcomes were (i) self-rated individual performance, (ii) supervisor-rated individual performance, (iii) self-rated team performance on both the level of the individual (How does a team member perceive the performance of his or her team?), and (iv) aggregated on the team level (How do the team members perceive their performance on average?), (v) supervisor-rated team performance, (vi) self-rated individual work satisfaction, (vii) self-rated teamwork quality on both the level of the individual (How does a team member perceive the teamwork quality in his or her team?), and (viii) aggregated on team level (How do the team members perceive their teamwork quality on average?). The outcomes are summarized in Table 1.

**TABLE 1:** Outcomes in the present study.

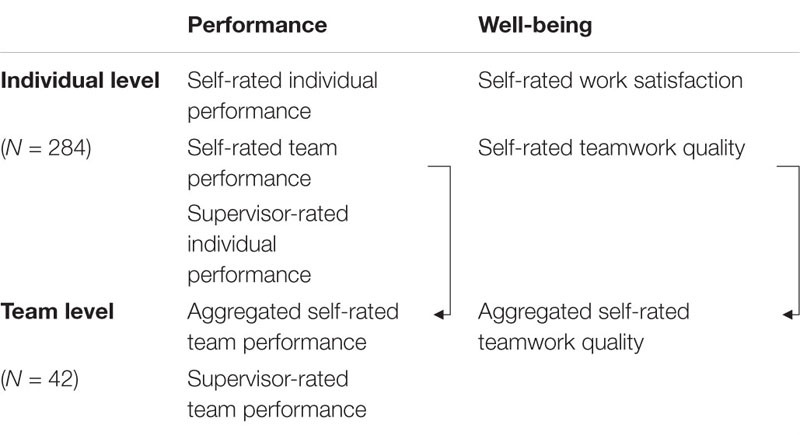

The present study had six main aims: first, we aimed at examining the relationships between current and ideal team roles and character strengths with work-related outcomes. Thereby, we intended to corroborate earlier findings on positive relationships of team roles (Gander et al., 2018; Ruch et al., 2018) and character strengths (e.g., Harzer and Ruch, 2014; Gander et al., 2020; Heintz and Ruch, 2020; Huber et al., 2020) with work-related outcomes and extending these findings by analyzing hitherto not studied outcomes, such as team performance and teamwork quality, and by additionally considering the team-level perspective. In line with previous findings, we expected positive relationships of all current team roles with work satisfaction, teamwork quality, and performance because enactment of these roles is considered conducive to achieving work tasks as well as to being satisfied with one’s work. For character strengths, we expected positive relationships of work satisfaction and teamwork quality with the strengths of teamwork, zest, love, curiosity, gratitude, and hope, and a positive association between performance and the strength of perseverance.

Second, we aimed at studying whether a good convergence between ideal and current team roles goes along with better outcomes. We examined this research question on both the level of individuals (i.e., whether the convergence between an individual’s ideal and current team role goes along with better outcomes), and the level of teams (i.e., whether teams with higher average levels of convergence between the team member’s ideal and current team roles report better outcomes). While earlier studies (Gander et al., 2018) analyzed the relationships of current-ideal convergence with job satisfaction and calling, no study has addressed the relevance of this convergence for performance, or on the level of the team. Based on the findings by Gander et al. (2018), we hypothesized higher levels of performance, work satisfaction, and teamwork quality for more convergent individuals and teams.

Third, we examined whether the number of team roles represented in the current team goes along with the outcomes. In line with theoretical assumptions for the VIA team roles (adapted from Belbin, 2010), we hypothesized higher levels in all outcomes in more balanced teams in which more of the team roles are represented.

Fourth, we studied for each team role separately, whether the outcomes are affected by the number of team members representing this role. In line with theoretical assumptions for the VIA team roles (adapted from Belbin, 2010), we expected that having multiple team members assuming the same roles might have detrimental effects on the outcomes (i.e., that the number of team members representing this role would be negatively related to the outcomes).

Fifth, we examined whether balance in teams with regard to character strengths (i.e., how many character strengths are represented in a team) also relates to the outcomes. This idea was examined on an exploratory basis, and we did not formulate specific hypotheses.

Finally, we tested for each character strength separately, whether there are detrimental effects on the outcomes, when a strength is represented by several team members. Based on theoretical considerations (Peterson and Seligman, 2004) and earlier empirical findings on the individual level for other outcomes, such as life satisfaction (Park et al., 2004) and calling (Harzer and Ruch, 2012), we expected that this is not the case and that there is no such thing as “too much” of a character strength, also with regard to teams. Thus, we conducted these analyses on an exploratory basis. The hypotheses and findings are summarized in Table 2.

**TABLE 2:** Overview over hypotheses and findings.

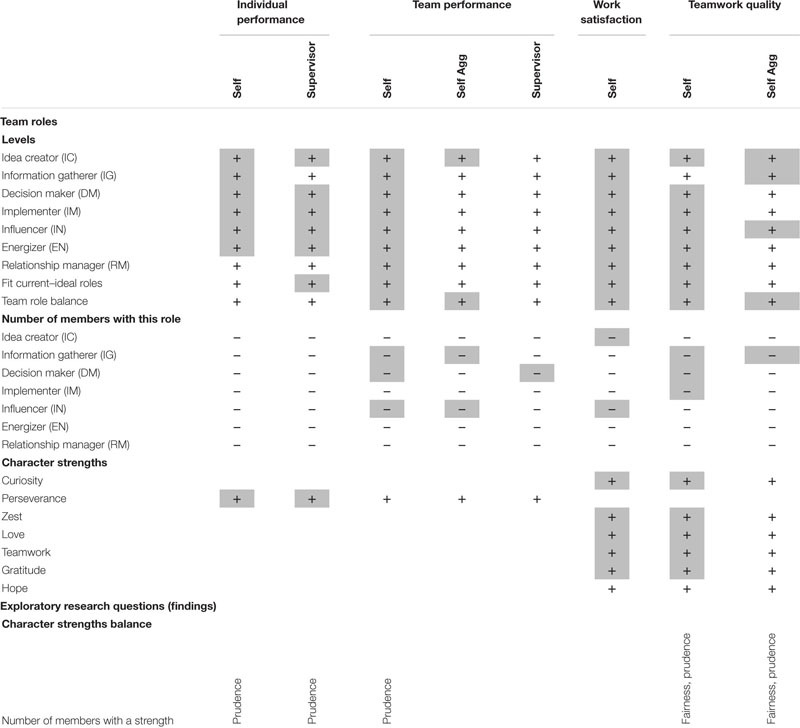

*Hypothesized positive effects are denoted by a plus sign (+), negative effects by a minus sign (−). Signs in green denote that the hypothesis was confirmed, signs and orange denote that the hypothesis was not confirmed. For exploratory research questions, no hypotheses were formulated, and only those strengths are given where significant effects were observed.*

## Materials and Methods

### Participants

#### Individuals

The sample of team members consisted of 284 (41.2% men) participants aged between 16 and 66 (*M* = 42.18, *SD* = 10.62). Most participants (69.4%) held a degree from a university or a university of applied sciences, 6.7% held a diploma allowing them to attend such universities, 19.4% completed vocational training, and 4.6% completed mandatory school. Most participants (82.7%) completed the German version of the survey; the remaining participants completed an English version. On average, participants had been working for *M* = 4.48 years (*SD* = 5.54 years) in the team, with a broad range from less than 1 year up to 34 years.

#### Teams

The 284 team members were working in *N* = 42 teams. Team sizes varied between 3 and 15 members (*M* = 8.49; *SD* = 3.25 members). Teams were from a broad array of occupations and sectors, including public administration (38.1%), international corporations (21.4%), health care (14.3%), technology and engineering (11.9%), education and research (7.1%), law firms (4.7%), and one team from the service sector.

#### Supervisors

The 42 teams were led by *N* = 42 supervisors (61.9% women) aged 28–62 (*M* = 47.31, *SD* = 9.18). These supervisors represented the direct supervisors and were not team members themselves but represent a separate sample.

### Instruments

The *VIA Team-Roles Inventory* (Ruch et al., 2018) assesses the degree to which one masterfully performs the seven VIA team roles (i.e., Idea Creator, Information Gatherer, Decision Maker, Implementer, Influencer, Energizer, and Relationship Manager) in the current team with five items each. Respondents read a short description of the roles and then are asked about their ability to perform this role, and their enjoyment and engagement/flow in performing this role. All items used a seven-point Likert-style scale, ranging from 1 (“strongly disagree”) through 7 (“strongly agree”). A sample item is, “In my current team, I’m at my best when coming up with ideas” (Idea Creator). Internal consistencies in the present study were high (all α ≥ 0.92).

The *VIA Ideal Team-Roles Inventory* (Gander et al., 2018) assesses the degree to which one would perform the seven VIA team roles in an ideal team. Participants were asked to think of an ideal team, i.e., a team in which they could apply all their strengths and do what they do best. All items used a seven-point Likert-style scale, ranging from 1 (“strongly disagree”) to 7 (“strongly agree”). A sample item is, “If I would be in my ideal team, I’d be at my best when coming up with ideas” (Idea Creator). Internal consistencies in the present study were high (all α ≥ 0.93).

The *Values in Action Inventory of Strengths* (VIA-IS; Peterson and Seligman, 2004; German version by Ruch et al., 2010) assesses the 24 character strengths of the VIA classification with 10 items per character strength. It uses a five-point Likert-style scale ranging from 5 (=“very much like me”) to 1 (=“very much unlike me”). A sample item is, “I find the world a very interesting place” (curiosity). Internal consistencies in the present study ranged from α = 0.68 to α = 0.91 (median α = 0.76).

The *Teamwork Quality Questionnaire* (TWQ; Hoegl and Gmuenden, 2001) assesses six facets of collaborative team process (i.e., communication, coordination, balance of member contributions, mutual support, effort, and cohesion) capturing both task-related and social interaction within teams with 38 items. The questionnaire uses a five-point Likert-style scale ranging from 1 (“strongly disagree”) through 5 (“strongly agree”). A sample item is “There was frequent communication within the team” (communication). In the present study, we only analyzed general teamwork quality (i.e., the total score across all items). Internal consistency was high (α = 0.95), and there was a good inter-rater reliability among team members (ICC [2]; one-way random effects, absolute agreement, average of multiple raters = 0.82), and there was a considerable amount of variance attributed to group membership (ICC [1] = 0.40). Inter-rater-agreement for the individual teams ranged from *r*_WG(J)_ = 0.96 to 0.99 [median *r*_WG(J)_ = 0.99].

For the assessment of *Work Satisfaction*, we selected the 11 items out of the 15 items suggested by Warr et al. (1979) that clearly loaded on a general job satisfaction factor and did not show secondary loadings in a previous study (Parker, 2000). All items are rated on a seven-point Likert-style scale ranging from 1 (“extremely dissatisfied”) to 7 (“extremely satisfied”). A sample item is, “How satisfied are you with the opportunity to use your ability?” Internal consistency was high (α = 0.87).

For the assessment of self- and supervisor-rated *Team Performance and Individual Performance*, we adapted five items suggested by Hoegl and Gmuenden (2001). The items for the assessment of team performance, rated both by each team member and the team supervisor, were: “Going by the results, the work of the team can be regarded as successful,” “The work of the team is of high quality,” “The team was satisfied with the results of the team’s work,” “The team achieves its goals,” and “The team completes its tasks within schedule.” Further, we adapted these five items for the assessment of self- and supervisor-rated work performance: “Going by the results, my work can be regarded as successful,” “My work is of high quality,” “I am satisfied with the results of my work,” “I achieve my goals,” and “I complete my tasks within schedule.” Internal consistencies were high (team performance self-rating: α = 0.87, team performance supervisor rating: α = 0.78, individual performance self-rating: α = 0.81, individual performance supervisor rating: α = 0.91), while inter-rater reliability for self-rated team performance was moderate (ICC [2] = 0.64), and 21% percent of the variance could be attributed to team membership (ICC [1]). Inter-rater agreement for the individual teams ranged from *r*_WG(J)_ = 0.79 to 0.99 [median *r*_WG(J)_ = 0.96].

### Procedure

According to the university’s ethics guidelines, no formal ethics proposal was needed for the present study. All data was collected online. We recruited participants via their supervisors who were contacted through professional networks, psychology mailing lists, psychology magazines, and meet-up groups. Individuals who are currently members of a work team of three or more people were eligible for participation. A work team is defined as a group of people that comprise a set of complementary skills and whose members interact with each other to achieve an—at least partially—common goal.

First, the team supervisor received a link to an online survey, asking for the e-mail addresses of all team members. The supervisors completed performance evaluations of the individual team members and the team as a whole. Afterward, each team member received an invitation to participate in an online survey in which they provided demographic information and completed the measures on character strengths, team roles, job satisfaction, teamwork quality, and individual and team performance. Before the start of the questionnaire, all supervisors and team members provided written informed consent. All questionnaires could be completed in German or English. Upon request, each participant received a feedback on his or her individual character strength profile and a team-based feedback on the team role balance, character strengths balance, and aggregated levels of teamwork quality. No other incentives for participation were offered.

### Data Analysis

#### Convergence Between Current and Ideal Team Roles

For computing an overall indicator of convergence between current and ideal team roles, we computed the Euclidian distance, that is, the square root of the sums of the squared differences between every current (VIA Team Roles Inventory) and ideal (VIA Ideal Team Roles Inventory) team role. The resulting indicator is a measure of discrepancy: lower scores denote a better convergence between ideal and current team roles. While earlier studies suggested more complex relationships between current and ideal team roles, also depending on the type of role (Gander et al., 2018), we used this measure as an overall indicator of convergence.

#### Team Role/Character Strength Balance

For studying the effects of balance with regard to team roles and character strengths, we computed two different types of indices: The first type of indices indicates how many of the seven team roles or the 24 character strengths are represented in a team. Thus, for every team role (and character strength), we determined that it was present in a given team, when at least one of the members scored among the highest 10% in this scale. For each team role (and character strength), the team received one point if the role/strength was present—regardless of how many team members represented the role/strength—and zero points if the role/strength was represented by none of the team members. This resulted in two overall balance indices for each team; one for team roles and one for character strengths. These indices ranged from 0 to 7 for team roles and from 0 to 24 for character strengths. The overall balance indices were used for determining whether individuals and teams are more satisfied and perform better when all roles are represented.

The second type of indices indicated by how many times a team role or character strength was represented by a team member. Thus, for each team member who represented the role/strength of interest, the team received one point. This resulted in seven indices for team roles, and 24 indices for character strengths, each ranging from 0 to the total number of team members. We tested for linear and quadratic trends in these indices, for examining whether there are negative effects on the outcomes when some roles are represented several times in a team. All analyses using these balance indices were controlled for the number of team members (team size).

#### Statistical Analyses

We had data on the team-level (Level 2; i.e., team size, gender ratio, average age of team members, average educational level of team members, average duration of team membership, average fit between ideal and current roles, supervisor ratings of team performance, and number of team roles/character strengths present in the team) and on the person-level (Level 1; i.e., gender, age, education, duration of team membership, fit between current and ideal roles, self-ratings of work satisfaction, individual performance, team performance, teamwork quality, and supervisor-ratings of individual performance), with the person-level nested within the team-level. We used the R-package lme4 (Bates et al., 2015) for analyzing multilevel models, and lmerTest (Kuznetsova et al., 2017) for computing *p*-values for the fixed effects. All models with Level 1 outcomes (i.e., predicting self- and supervisor-rated individual performance, self-rated team performance, work satisfaction, and teamwork quality) were estimated using a restricted maximum likelihood estimation and allowed random intercepts for the teams. Since preliminary analyses suggested relationships of several demographic variables (e.g., gender and education) and objective team characteristics (e.g., gender ratio and average education level) with the outcomes, we controlled all subsequent analyses for team size, as well as individual and team-level gender, age, education, and duration of team membership.

The only exceptions were the analyses with supervisor-rated team performance as outcome (Level 2). For these analyses, we computed ordinary least squares regressions using only aggregated Level 2 data as predictors and control variables (i.e., team size, gender ratio, average age, average education, and average duration of team membership).

## Results

Zero-order correlations between all variables in the study on both the individual level, and on the aggregated team-level are given in online Supplementary Table A.

### Levels of Current and Ideal Team Roles

First, we inspected the relationships between the levels of current and ideal team roles with the outcomes by computing a set of multilevel models predicting the outcomes by each team role separately, and the control variables (see Table 3).

**TABLE 3:** The relationship of current and ideal team role levels with the outcomes.

	**Individual performance**		**Team performance**			**Work satisfaction**	**Teamwork quality**	
	**Self**	**Supervisor**	**Self**	**Self Agg**	**Supervisor**	**Self**	**Self**	**Self Agg**
**Current roles**								
IC	0.22***	0.22***	0.20**	0.52**	0.15	0.43***	0.24***	0.69**
IG	0.13*	0.10	0.14*	0.29	–0.04	0.19***	0.10	0.54**
DM	0.21**	0.16**	0.13*	0.20	0.08	0.27***	0.12*	0.39
IM	0.25***	0.19**	0.20**	0.32	0.05	0.25***	0.14**	0.35
IN	0.18**	0.21***	0.17**	0.35*	0.02	0.26***	0.21***	0.47*
EN	0.13*	0.16**	0.16**	0.22	–0.07	0.29***	0.15**	0.30
RM	0.08	0.11	0.18**	0.22	–0.18	0.33***	0.20***	0.34
**Ideal roles**								
IC	0.14*	0.09	0.03	0.11	0.11	0.11	0.00	0.05
IG	0.11	0.07	0.08	0.23	–0.01	0.03	–0.05	0.26
DM	0.21*	0.09	0.05	0.06	–0.10	0.02	–0.01	–0.04
IM	0.22*	0.07	0.07	0.22	0.00	0.11	0.05	0.32
IN	0.16*	0.12*	0.11	0.24	–0.12	0.08	0.06	0.16
EN	0.09	0.15*	0.04	–0.28	–0.14	0.13*	0.02	–0.31
RM	0.10	0.01	0.08	0.09	–0.21	0.13*	0.11*	0.03

N_*Individual*_ = 277–284; N_*Teams*_ = 36–42 (for aggregated self-reported team performance and teamwork quality, and supervisor-rated team performance). Self Agg, Team-level aggregated self-reports; IC, Idea creator; IG, Information gatherer; DM, Decision maker; IM, Implementer; IN, Influencer; EN, Energizer; RM, Relationship Manager. All coefficients are standardized fixed effects from multilevel level models. All analyses with Level-1 outcomes (all self-ratings and supervisor ratings of individual performance) were controlled for team size and individual and team-level gender, age, education, and duration of team membership. Analyses with Level-2 outcomes (all aggregated ratings, and supervisor-ratings of team performance) were controlled for team size, gender ratio, average age, average education, and average duration of team membership.*p < 0.05, **p < 0.01, ***p < 0.001.

Table 3 shows that most current team roles positively related to self- and supervisor-rated individual performance (exceptions were Information Gatherer and Relationship Manager), and to self-rated team performance, but not supervisor-rated team performance. Overall, the numerically strongest relationships were found for the Idea Creator and Implementer roles. All seven team roles contributed to individual work satisfaction, while all roles but Information Gatherer related to self-rated teamwork quality. At the team-level, higher average levels of Idea Creator, Information Gatherer, and Influencer were associated with higher average scores of teamwork quality.

Only a few relationships were found for the levels of ideal roles. Some roles were related to self- (Idea Creator, Decision Maker, Implementer, and Influencer) or supervisor-rated (Influencer and Energizer) individual performance, work satisfaction (Energizer and Relationship Manager), or self-rated teamwork quality (Relationship Manager), while all roles were unrelated to supervisor-rated team performance.

### Convergence Between Current and Ideal Team Roles

For analyzing the relevance of the convergence between current and ideal team roles, we computed a set of multilevel models, predicting the outcomes by the indicator of convergence, and the control variables. Results are given in Table 4.

**TABLE 4:** The relationships of discrepancy between current and ideal team roles and team role balance with self- and supervisor-rated performance, work satisfaction, and teamwork quality.

	**Fit current-ideal roles**	**Team role balance**
**Individual performance**		
Self	–0.10	0.05
Supervisor	−0.16**	0.10
**Team performance**		
Self	−0.17**	0.38***
Self-aggregated	–0.26	0.65***
Supervisor	0.09	0.16
**Work satisfaction**	−0.31***	0.26**
**Teamwork quality**		
Self	−0.18***	0.49***
Self aggregated	–0.34	0.73***

N_*Individual*_ = 277–284; N_*Teams*_ = 36–42 (for aggregated self-reported team performance and teamwork quality, and supervisor-rated team performance). Fit Current-Ideal Roles, discrepancy (Euclidian distance) between current and ideal team roles. Team Role Balance, index of how many of the seven team roles are represented in a team. All coefficients are standardized fixed effects from multilevel level models. All analyses with Level-1 outcomes (all self-ratings and supervisor ratings of individual performance) were controlled for team size and individual and team-level gender, age, education, and duration of team membership. Analyses with Level-2 outcomes (all aggregated ratings and supervisor-ratings of team performance) were controlled for team size, gender ratio, average age, average education, and average duration of team membership.**p < 0.01, ***p < 0.001.

Table 4 shows that with regard to outcomes on the level of individuals, the smaller the discrepancy between current and ideal roles, the higher the supervisor-rated—but not self-rated—performance, and the higher the self-rated work satisfaction and perceived teamwork quality. On the level of teams (i.e., using aggregated outcomes), no effects of current/ideal-convergence were observed.

### Team Role Balance

The index of team role balance ranged between 0 and 7, with an average of *M* = 4.31 roles (*SD* = 2.23) represented in each team. For analyzing the effects of team role balance, we computed the same analyses, predicting the outcomes by the team role balance and the control variables.

Table 4 shows that the more the seven VIA team roles are represented in each team, the better the self-rated team performance. Further, the number of team roles represented also went along with higher reported work satisfaction and teamwork quality. No relationship was found for supervisor-rated individual performance. On the level of teams, the number of team roles represented showed positive effects on self-rated team performance and teamwork quality.

Further, for each team role, we looked at how many times they were represented in a team. These indices ranged from the minimum of 0 (for all team roles) to the maxima of 4 (Idea Creator, Information Gatherer, and Relationship Manager), 6 (Energizer), 7 (Decision Maker and Implementer), and 8 (Influencer) persons in a team representing these roles. Averages ranged from *M* = 0.76 roles (Information Gatherer) to *M* = 1.64 roles (Implementer) with standard deviations between *SD* = 0.96 (Information Gatherer) and *SD* = 1.45 (Influencer).

For examining whether there is a satiation point of the number of people representing a team role, we computed a set of multilevel models, and estimated both linear and quadratic trends. Thus, we predicted the outcomes by the number of team members representing this role, and the squared number of team members representing this role (predictors were mean-centered for avoiding issues of multicollinearity), and the control variables. Results are given in Table 5.

**TABLE 5:** The relationships of the number of team roles represented in each team with self- and supervisor-rated performance, work satisfaction, and teamwork quality.

	**Individual performance**		**Team performance**			**Work satisfaction**	**Teamwork quality**	
**Number of members**	**Self**	**Supervisor**	**Self**	**Self agg**	**Supervisor**	**Self**	**Self**	**Self agg**
**IC**								
Linear	0.10	0.20	0.41**	0.61*	0.16	0.39***	0.64***	0.81***
Quadr	–0.06	–0.27	–0.29	–0.16	–0.25	−0.24*	–0.36	–0.25
**IG**								
Linear	0.03	0.04	0.45***	0.80***	0.07	0.32**	0.62***	0.85***
Quadr	–0.01	–0.11	−0.36**	−0.47*	–0.21	–0.20	−0.38*	−0.38*
**DM**								
Linear	–0.04	0.16	0.57**	0.58**	0.55*	0.23	0.66**	0.56**
Quadr	0.01	–0.26	−0.46**	0.04	−0.52**	–0.18	−0.49*	–0.02
**IM**								
Linear	0.12	0.09	0.39*	0.56**	0.07	0.12	0.41*	0.44
Quadr	0.03	–0.15	–0.19	0.14	–0.28	–0.02	–0.16	0.04
**IN**								
Linear	0.18	0.32*	0.43*	0.42*	0.54**	0.22	0.54*	0.50**
Quadr	–0.09	−0.33*	−0.36*	–0.01	−0.54**	–0.15	–0.42	–0.26
**EN**								
Linear	0.14	–0.06	0.22	0.32	0.05	0.21*	0.18	0.21
Quadr	–0.07	–0.03	–0.20	–0.16	–0.23	–0.11	–0.13	0.10
**RM**								
Linear	0.03	0.19	0.20	0.25	0.19	0.27**	0.29	0.44
Quadr	0.06	–0.21	–0.10	0.02	–0.32	–0.11	–0.16	0.16

N_*Individual*_ = 277–284; N_*Teams*_ = 36–42 (for aggregated self-reported team performance and teamwork quality, and supervisor-rated team performance). Self Agg, Team-level aggregated self-reports; No. of members, How many members of a team represented the role of interest; Linear, Linear effects; quadratic, Quadratic effects; IC, Idea creator; IG, Information gatherer; DM, Decision maker; IM, Implementer; IN, Influencer; EN, Energizer; RM, Relationship Manager; Linear, linear relationships; quadr, quadratic relationships. All coefficients are standardized fixed effects from multilevel level models. All analyses with Level-1 outcomes (all self-ratings and supervisor ratings of individual performance) were controlled for team size and individual and team-level gender, age, education, and duration of team membership. Analyses with Level-2 outcomes (all aggregated ratings, and supervisor-ratings of team performance) were controlled for team size, gender ratio, average age, average education, and average duration of team membership.*p < 0.05, **p < 0.01, ***p < 0.001.

**FIGURE 1:**
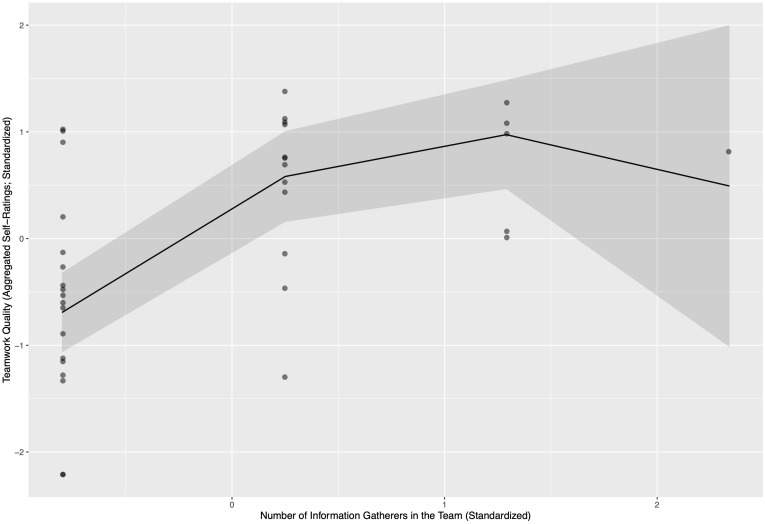
Relationship of the number of information gatherers per team and teamwork quality (standardized coefficients).

Table 5 shows that for individual performance, there were only effects for the team role of influencer (Influencer): Results suggested an inverted u-shape relationship between the number of people representing the role of influencer and the supervisor-rated individual performance. Figure 1 shows an example of the nature of this u-shaped relationship.

Similar patterns were also observed for self-rated team performance (for the roles of Information Gatherer, Decision Maker, and Influencer), while for the roles of Idea Creator and Implementer, only a positive linear effect was observed, while the quadratic effects did not reach significance. For work satisfaction, again, inverted u-shaped relationships were found for Idea Creator, while linear effects were obtained for Information Gatherer, Energizer, and Relationship Manager roles. For teamwork quality, u-shaped relationships were found for Information Gatherer and Decision Maker, and linear relationships for Idea Creator and Influencer. Finally, on the level of teams, we found the same linear and quadratic effects for the roles of Decision Maker and Influencer for supervisor-rated team performance. Further, aggregated self-ratings were mostly parallel to the findings for individual self-ratings.

### Levels of Character Strengths

As for team roles, we computed a set of multilevel models predicting the outcomes by the level of each character strength separately and the control variables (see Table 6).

**TABLE 6:** The relationship of character strength levels with self- and supervisor-rated performance, work satisfaction, and teamwork quality.

	**Individual performance**		**Team performance**			**Work satisfaction**	**Teamwork quality**	
	**Self**	**Supervisor**	**Self**	**Self agg**	**Supervisor**	**Self**	**Self**	**Self agg**
Creativity	0.12	0.02	–0.02	0.18	0.08	–0.02	–0.03	0.01
Curiosity	0.14*	0.01	0.10	0.36	–0.17	0.12*	0.21***	0.37
Judgment	0.12	0.06	0.03	0.37	0.07	0.00	0.01	0.33
Learning	–0.01	–0.01	0.04	0.35	0.00	–0.02	0.05	0.36
Perspective	0.25***	0.04	0.06	0.06	–0.12	0.01	0.03	–0.05
Bravery	0.08	–0.04	0.02	0.07	–0.04	–0.01	0.00	–0.07
Perseverance	0.34***	0.11*	0.10	0.22	–0.05	0.09	0.00	–0.08
Honesty	0.23***	0.00	0.13*	0.31	–0.20	0.11	0.07	0.18
Zest	0.23***	0.01	0.09	–0.07	–0.04	0.15*	0.10*	–0.20
Love	0.16*	0.01	0.16**	0.15	0.11	0.22***	0.19***	0.09
Kindness	0.16*	0.00	0.14*	0.06	–0.15	0.16**	0.14**	0.02
Social intelligence	0.14*	–0.01	0.07	0.21	–0.07	0.13*	0.15**	0.11
Teamwork	0.15*	0.04	0.24***	0.34	0.00	0.37***	0.28***	0.47**
Fairness	0.07	0.05	0.16**	0.25	0.01	0.16**	0.16**	0.35*
Leadership	0.25***	0.05	0.11*	0.05	–0.08	0.13*	0.09	0.02
Forgiveness	0.05	0.02	0.06	0.29	–0.11	0.19**	0.11*	0.32
Humility	0.07	0.03	0.00	0.15	–0.03	0.07	0.00	0.38
Prudence	0.12	0.05	0.07	0.15	0.00	0.12*	0.11*	0.11
Self-regulation	0.24***	0.04	0.06	–0.12	0.21	0.10	0.12*	–0.22
ABE	0.10	–0.05	0.05	0.25	0.02	0.05	0.09	0.22
Gratitude	0.21**	0.00	0.12*	0.17	–0.05	0.22***	0.13*	0.12
Hope	0.25**	0.07	0.05	–0.04	–0.11	0.11	0.08	–0.23
Humor	0.05	–0.04	0.02	0.01	–0.20	0.01	–0.01	000
Spirituality	0.02	–0.06	0.02	–0.20	0.01	0.02	0.12	–0.28

N_*Individual*_ = 277–284; N_*Teams*_ = 36–42 (for aggregated self-reported team performance and teamwork quality, and supervisor-rated team performance). Self Agg, Team-level aggregated self-reports; Learning, Love of learning; ABE, Appreciation of beauty and excellence. All coefficients are standardized fixed effects from multilevel level models. All analyses with Level-1 outcomes (all self-ratings and supervisor ratings of individual performance) were controlled for team size and individual and team-level gender, age, education, and duration of team membership. Analyses with Level-2 outcomes (all aggregated ratings, and supervisor-ratings of team performance) were controlled for team size, gender ratio, average age, average education, and average duration of team membership. All coefficients are standardized fixed effects.*p < 0.05, **p < 0.01, ***p < 0.001.

Table 6 shows that several character strengths (including perseverance, perspective, leadership, hope, self-regulation, honesty, zest, and gratitude) predicted self-rated individual performance; only perseverance was associated with supervisor-rated individual performance. A similar picture was obtained for team performance, where several character strengths were associated with self-rated individual team performance (mostly teamwork, love, and fairness), but no strengths were related to supervisor-rated or aggregated self-rated team performance. Work satisfaction and teamwork quality were predicted by several character strengths (strongest relationships for teamwork and love) in self-ratings, while on the level of teams, only teamwork and fairness were significant predictors of teamwork quality.

### Character Strength Balance and Number of Character Strengths Represented

We computed the same analyses for character strengths as for team roles, for examining whether the character strength balance, that is, how many of the 24 character strengths of the VIA classification are represented in each team, relate to the outcomes. Between 3 and 24 of the character strengths were represented in each team (*M* = 13.31; *SD* = 5.84). Results are given in Table 7.

**TABLE 7:** The relationships of the character strengths balance and the number of character strengths represented in each team with self- and supervisor-rated performance, work satisfaction, and teamwork quality.

	**Individual performance**		**Team performance**			**Work satisfaction**	**Teamwork quality**	
	**Self**	**Supervisor**	**Self**	**Self agg**	**Supervisor**	**Self**	**Self**	**Self agg**
Character strengths balance	0.09	0.12	0.02	–0.01	0.25	–0.04	0.07	0.07
**No. of members**								
Creativity	0.06	–0.02	–0.11	–0.16	–0.16	–0.02	–0.12	–0.19
Curiosity	0.14	0.13	0.08	0.13	0.15	0.05	0.09	0.12
Judgment	0.09	0.04	0.15	0.24	0.10	0.01	0.14	0.23
Learning	0.08	0.02	0.04	0.07	0.00	0.04	0.08	0.10
Perspective	0.03	0.02	0.11	0.16	0.12	–0.04	0.20	0.24
Bravery	0.13	0.01	0.03	0.07	0.15	–0.14	–0.06	–0.07
Perseverance	0.11	–0.11	–0.06	–0.02	–0.09	–0.01	–0.02	0.01
Honesty	0.12	0.00	0.05	0.07	0.00	0.10	0.10	0.14
Zest	–0.02	0.11	–0.01	–0.02	0.20	–0.07	–0.03	–0.05
Love	0.06	0.15	0.10	0.11	0.19	0.01	0.08	0.09
Kindness	0.06	–0.02	0.12	0.17	–0.07	0.05	0.12	0.16
Social intelligence	–0.05	0.06	0.06	0.08	0.16	–0.08	0.03	0.07
Teamwork	0.05	–0.09	0.12	0.20	–0.18	0.08	0.21	0.30
Fairness	0.07	0.13	0.18	0.30	0.14	0.03	0.28*	0.39*
Leadership	0.08	–0.01	0.16	0.26	0.15	–0.02	0.14	0.20
Forgiveness	0.09	0.05	0.17	0.31	0.06	0.02	0.14	0.23
Humility	0.20	–0.17	0.20	0.39	0.04	0.03	0.24	0.39
Prudence	0.28**	0.11	0.35**	0.60**	0.23	0.07	0.34*	0.45*
Self-regulation	0.16	–0.03	0.01	0.06	0.12	–0.10	–0.14	–0.16
ABE	–0.01	0.03	–0.02	–0.02	0.09	–0.01	0.05	0.08
Gratitude	–0.06	0.05	0.06	0.07	0.02	0.01	0.08	0.14
Hope	0.08	0.04	–0.05	–0.11	0.18	–0.04	–0.15	–0.19
Humor	–0.01	–0.03	0.08	0.16	0.13	–0.03	0.10	0.17
Spirituality	0.00	0.01	–0.13	–0.21	–0.07	–0.09	–0.08	–0.15

N_*Individual*_ = 277–284; N_*Teams*_ = 36–42 (for aggregated self-reported team performance and teamwork quality, and supervisor-rated team performance). Self Agg, Team-level aggregated self-reports; Character Strengths Balance, Index of how many of the 24 character strengths are represented in a team; No. of members, How many members of a team represented the character strength of interest; Learning, Love of learning; ABE, Appreciation of beauty and excellence. All coefficients are standardized fixed effects from multilevel level models. All analyses with Level-1 outcomes (all self-ratings and supervisor ratings of individual performance) were controlled for team size and individual and team-level gender, age, education, and duration of team membership. Analyses with Level-2 outcomes (all aggregated ratings, and supervisor-ratings of team performance) were controlled for team size, gender ratio, average age, average education, and average duration of team membership.*p < 0.05, **p < 0.01.

Table 7 shows that no relationships were observed between character strength balance and the outcomes.

Next, we analyzed whether the number of members in each team representing each of the 24 character strengths relates to the outcomes. Since analyses suggested no quadratic effects of character strengths, only linear effects were examined. Only for the character strengths of fairness (positive relationships with teamwork quality) and prudence (positive relationships with self-rated individual and team performance and teamwork quality) effects were observed.

## Discussion

The present study examined the contributions of team roles and character strengths to well-being and performance at work on both the levels of individuals and teams. Overall, our expectations were mostly confirmed for self-ratings of the outcomes, while they only were partially confirmed for supervisor ratings and team-level aggregated self-ratings. On the level of teams, this can mostly be explained by insufficient power due to the small sample size on the level of teams, since most effects were in the expected direction but failed to reach significance. Also, especially in the supervisor ratings, there was less variance, and potential relationships might be hidden by ceiling effects. Nonetheless, it is also possible that self-ratings of performance (on individual and team-level) assess somewhat different constructs than supervisor ratings and that the former are more strongly influenced by perceptions of teamwork quality and satisfaction than the latter. In the following, we summarize and discuss our main findings.

### Team Roles

#### Effects of Team Roles on Performance, Work Satisfaction, and Teamwork Quality

First, higher levels in most current team roles—but not ideal team roles—went along with higher levels of work satisfaction and teamwork quality, individual performance (both self- and supervisor-rated), and self-rated team performance, thus, widely confirming our expectations. On the level of teams, however, although the effects of self-ratings on team performance and teamwork quality went into the expected direction, only a few effects reached significance, and no relationships with supervisor-rated team performance were observed. Since these analyses were performed at the level of teams, the statistical power was determined by the sample size of teams and was likely not sufficient to detect the effects—even though the sample size of teams was considerably larger than in many previous studies. Compared to the other team roles, Information Gatherer and Relationship Manager seemed to be least important for performance, and Information Gatherer for well-being at work, while the most robust results across all outcomes were found for Idea Creator. One might argue that this is due to the sample that consisted mostly of higher-level occupations where coming up with new, innovative approaches is a core requirement of the job, while gathering information might be considered a more basic skill that several people should be able to perform, and that is therefore less appreciated.

#### Convergence Between Current and Ideal Roles

Further, a better convergence between current and ideal team roles went along with higher work satisfaction and better teamwork quality, thus confirming previous findings (Gander et al., 2018) and our expectations. For individual and team performance, we found some support for positive relationships, although they did not show up in all different data sources and levels of analysis considered. Nonetheless, we conclude that increasing the convergence between current and ideal roles might offer a valuable starting point for interventions aimed at fostering individual work satisfaction. Although team roles represent *informal* roles that cannot be assigned, one still might consider ways to craft someone’s job in order to increase the fit to his or her ideal team role (Wrzesniewski and Dutton, 2001). Further research is needed on formal roles that facilitate the display of team roles; based on such information team roles might also be considered in selection procedures, for maximizing the person-job fit.

#### Team Role Balance

Team role balance showed the expected positive relationships to work satisfaction, teamwork quality, and performance on the level of teams; no effects were observed for supervisor ratings. Thus, how many of the seven VIA team roles are represented in a team is an important information for the well-being of the team members, although this does not necessarily translate to effects on performance that could also be perceived by external evaluators, such as the team supervisor. Nonetheless, a satisfying work experience can be considered an important factor for attracting and retaining employees (e.g., Michaels et al., 2001). Therefore, designing teams with the intention to have all team roles represented could be a helpful endeavor for the benefit of both the individual and the organization. In the present study, the operationalization of team role balance allowed each team member to represent multiple roles; thus, a balanced team of five members can theoretically consist of one member representing all seven roles and four members representing no roles at all. It is up to future studies to examine whether the degree to which the team roles are evenly distributed among the members also plays a role—one might expect that this is indeed the case, and that it is beneficial for a team when all individuals contribute to the representation of the roles in the team.

Further, the study provided some evidence on the question whether having more team members assuming the role goes along with positive or detrimental effects. Results suggest a complex relationship: For several roles (i.e., Information Gatherer, Decision Maker, and Influencer), quadratic relationships between the number of team members with this role and team performance, and teamwork quality was found, suggesting that while it is beneficial to have some team members in this role, there is also a maximum that should not be surpassed in order to avoid detrimental effects. For Idea Creator and Implementer roles, mostly linear effects were found, while there were also trends for quadratic effects that did not reach significance, however. The number of Energizers and Relationship Managers showed the weakest relationships to the outcomes. Thus, we tentatively conclude that when designing teams, one should particularly pay attention to avoid an overrepresentation of Information Gatherer, Decision Maker, and Influencer roles. One possible reason for these effects might be that, on the one hand, these roles might be more prone to competition and rivalry that lead to internal conflicts when assumed by several members of a team. On the other hand, having more people to create and implement ideas might be beneficial since these roles could often be more directly related to the success of the team and go along with mutual inspiration. However, at this point, we can only speculate about possible processes; more information on the processes and mechanisms of team role and character strength balance is desirable. For example, conflicts might also trigger reflection and contribute to team learning (e.g., Schley and van Woerkom, 2014).

How many of these roles are to be considered an overrepresentation, however, cannot be answered by this study. In the present study, we controlled for the effects of team size in all our analyses. However, one might assume that this strongly depends on the team size, and larger teams might be able to need or accommodate more people with Decision Maker roles without detrimental effects, while for very small teams, one person might be enough.

### Character Strengths

#### Effects of Character Strengths on Performance, Work Satisfaction, and Teamwork Quality

The present study also underlined the relevance of character strengths for work-related outcomes. Our findings were in line with previous studies (e.g., Heintz and Ruch, 2020) with regard to the contributions of strengths such as love, gratitude, zest, and curiosity for work satisfaction, and also teamwork quality. However, the strengths of teamwork and fairness also contributed to both variables, and were the only two strengths that yielded significant effects on teamwork quality on the team level. Both strengths also yielded the highest numerical relationships to self-rated team performance, which is in line with findings on the relationships of character strengths with students’ performance in group work (Wagner et al., 2020b). For individual performance, perseverance was found to be most important and related to both self- and supervisor-rated performance, in line with earlier findings. Thus, we conclude that perseverance is the single most relevant strength when interested in maximizing individual performance in selection decisions (in line with earlier findings; Harzer and Ruch, 2014; Littman-Ovadia and Lavy, 2016), while teamwork and fairness should be considered when selecting employees for tasks involving high amounts of cooperation in order to expect high levels of well-being in the teams.

#### Character Strength Balance

When looking at configurations of character strengths in teams, no support was found for the idea that all character strengths should be present in a team for all considered outcomes. One might assume that while some character strengths are highly relevant to work-related behavior and experiences in most occupations (i.e., persistence) several other character strengths are of lesser relevance in many occupations (e.g., spirituality, appreciation of beauty and excellence). However, one would also expect variation among jobs regarding the character strengths of most relevance (see e.g., Heintz and Ruch, 2020). Thus, not all 24 strengths of the VIA classification might be relevant in all jobs; in future studies, one might consider determining in a first step how many character strengths are potentially relevant in a particular team and examining in a second step whether those teams in which all relevant character strengths are represented outperform teams in which only few relevant strengths are represented.

Also, in line with our expectations, we found no evidence for detrimental effects when there are many team members with the same character strength in a team. This supports the idea that character strengths represent positive characteristics and that there is no such thing as having too much (or, in this case, too many) of a character strength. For two strengths, we found positive (linear) relationships between some outcomes and the number of people with the strengths in the team: this was the case for the strengths of prudence (self-rated individual and team performance, teamwork quality) and fairness (teamwork quality). This is especially interesting, since these relationships were also observable on the team levels: Thus, teams with more people who score high in prudence or fairness report better functioning. Both these character strengths might help in preventing conflicts within the team (i.e., being more careful in one’s actions and treating other members just). As opposed to team roles, having multiple members with these strengths might not lead to conflicts due to rivalry but instead could allow for a mutual support.

Although these findings should not be overinterpreted due to the large number of comparisons, they underline the relevance of character strengths such as fairness and prudence that are often overlooked or considered of lesser relevance when only positive outcomes on the individual level are considered (see e.g., Wagner et al., 2020a).

### Limitations

Of course, several limitations of the present study have to be addressed. First, the sample size of the teams was relatively small and only allowed for the detection of medium to large effects. Further, the present study pursued a quasi-experimental approach and studied real, existing teams. While studying real teams also represents the strength of the current study, no conclusions about directionality or causality of the findings can be made. Studies using experimental assignments of team roles or intervention studies aiming at changing team role behavior and/or balance are warranted that would allow for looking at causal influences of team roles on the outcomes. Further, most effects were found for self-reports that are prone to biases. While we also considered supervisor ratings for the performance-related outcomes, these ratings showed a slight negative skew and a restricted range. This limited variability in the supervisor-ratings might have led to an underestimation of the relationships. Also, one might argue that information from peers on the team members’ assumed team roles might also be considered for providing an additional perspective—in many teams, other team members might be able to provide a more accurate picture of a team member’s contributions than the supervisors who interact less frequently with the team members. Thus, future studies might also consider additional data sources. Finally, for examining the effects of team role and character strengths balance, we computed one index for counting how many of the seven team roles/24 character strengths are represented in a team, and indices for determining the number of roles/strengths represented by each team member. These indices rely on cutoff scores that were empirically derived for the present study; of course, such cutoff scores are always somewhat arbitrary and drastically reduce the amount of available information. Also, one might argue that different cutoffs for every team role/character strength would yield stronger effects—it is possible that for some roles/strengths, relatively low levels suffice for a team to function well, while for other roles/strengths, higher levels are needed. Thus, it is possible that a more sophisticated approach for measuring team role/character strength balance might yield even larger effects regarding the studied outcomes.

## Conclusion

In summary, the present study corroborated earlier research on the relationships of the VIA team roles and the convergence between current and ideal team roles with work satisfaction. Further, earlier findings of the relationships between character strengths and work satisfaction and performance were widely replicated. Additionally, we extended previous findings on team roles in the following main aspects: (1) The VIA team roles go along with better self- and supervisor-rated individual performance, and self-rated teamwork quality; (2) a better fit between current and ideal roles goes along with better supervisor-rated performance; (3) teams in which more team roles are represented report higher team performance and teamwork quality, both on the levels of individual and aggregated ratings; and (4) having too many team members sharing the same team role can go along with reduced levels of team performance and teamwork quality.

Further, previous research on character strengths was extended by also considering the team level: (5) We found that teams with higher average levels of teamwork or fairness report higher teamwork quality; (6) teams with more members with high levels in prudence or fairness report better teamwork quality and aggregated self-ratings of team performance (only prudence); and (7) there is no evidence that having too many members with high levels in a particular strength goes along with negative effects. We conclude that extending the study of character to the level of social systems, such as teams, provides a highly relevant new perspective, and more studies should examine the effects of different configurations of character strengths in such systems.

## Data Availability Statement

The raw data supporting the conclusions of this article will be made available by the authors, without undue reservation, to any qualified researcher.

## Ethics Statement

Ethical review and approval was not required for the study on human participants in accordance with the local legislation and institutional requirements. The patients/participants provided their written informed consent to participate in this study.

## Author Contributions

FG, IG, and WR conceptualized and designed the work. FG and IG analyzed and interpreted the data. FG drafted the article. FG and IG made critical revisions of the article. All authors contributed to the article and approved the submitted version.

## Conflict of Interest

WR is a Senior Scientist at the VIA Institute on Character, which holds the copyright of the VIA-IS and this study has been supported by the Manuel D. and Rhoda Mayerson Foundation and the VIA Institute on Character. The remaining authors declare that the research was conducted in the absence of any commercial or financial relationships that could be construed as a potential conflict of interest.

## Supplementary Material

FIGURE S1 Zero-order correlations among all study variables, on individual level (above diagonal) and team level (below diagonal). Above diagonal: correlations based on individual data (*N* = 277–284). Below diagonal: correlations based on aggregated team-level data (*N* = 36–42). ^∗^*p* < 0.05, ^∗∗^*p* < 0.01, ^∗∗∗^*p* < 0.001.
